# Irisin: An anti-inflammatory exerkine in aging and redox-mediated comorbidities

**DOI:** 10.3389/fendo.2023.1106529

**Published:** 2023-02-10

**Authors:** Caio dos Santos Trettel, Bruno Rocha de Avila Pelozin, Marcelo Paes Barros, André Luis Lacerda Bachi, Pedro Gabriel Senger Braga, César Miguel Momesso, Guilherme Eustáquio Furtado, Pedro Afonso Valente, Edilamar Menezes Oliveira, Eef Hogervorst, Tiago Fernandes

**Affiliations:** ^1^Interdisciplinary Program in Health Sciences, Institute of Physical Activity Sciences and Sports, Cruzeiro do Sul University, Sao Paulo, Brazil; ^2^Laboratory of Biochemistry and Molecular Biology of Exercise, School of Physical Education and Sport, University of Sao Paulo, Sao Paulo, Brazil; ^3^Post-Graduation Program in Health Sciences, Santo Amaro University, Sao Paulo, Brazil; ^4^Laboratory of Metabolism and Lipids, Heart Institute, University of Sao Paulo Medical School, Sao Paulo, Brazil; ^5^ENAU College, Innovation and Research Center, Sao Paulo, Brazil; ^6^Applied Research Institute, Polytechnic Institute of Coimbra, Coimbra, Portugal; ^7^Research Unit for Sport and Physical Activity (CIDAF, UID/PTD/04213/2020), Faculty of Sport Sciences and Physical Education (FCDEF-UC), Coimbra, Portugal; ^8^Research Centre for Sport and Physical Activity, Faculty of Sport Science and Physical Education, University of Coimbra, Coimbra, Portugal; ^9^Center for Neuroscience and Cell Biology, University of Coimbra, Coimbra, Portugal; ^10^National Centre for Sports and Exercise Medicine, Loughborough University, Loughborough, United Kingdom

**Keywords:** skeletal muscle, exercise, exerkines, myokines, oxidative stress, inflammaging

## Abstract

Human beings lead largely sedentary lives. From an evolutionary perspective, such lifestyle is not beneficial to health. Exercise can promote many enabling pathways, particularly through circulating exerkines, to optimize individual health and quality of life. Such benefits might explain the protective effects of exercise against aging and noncommunicable diseases. Nevertheless, the miRNA-mediated molecular mechanisms and exerkine interorgan crosstalk that underlie the beneficial effects of exercise remain poorly understood. In this mini review, we focused on the exerkine, irisin, mainly produced by muscle contraction during adaptation to exercise and its beneficial effects on body homeostasis. Herein, the complex role of irisin in metabolism and inflammation is described, including its subsequent effects on thermogenesis through browning to control obesity and improve glycemic regulation for diabetes mellitus control, its potential to improve cognitive function (via brain derived neurotrophic factor), and its pathways of action and role in aging.

## 1 Introduction

Despite millions of years of human evolution, the intricate genetic mechanisms and transposable chromosome elements have not adapted to the average low-level physical activity performed by modern *Homo sapiens sapiens* (1). Our ancestors, such as hunter-gatherers, engaged in intense physical activity to search for food and evade predators (2). As a result, physical activity was performed up to 6-9 hours per day in South Africa and Ache in Paraguay, including walking and running (3). According to prior studies, men in the Tsimane group engaged in 120 min/day of moderate or vigorous physical activity while women engaged in 100 min/day of such activity (4). Modern society has introduced human beings to a sedentary lifestyle. Currently, human beings spend hours seated in front of overbright screens, avoid using the stairs, even to go two floors up, and prefer the delivery of food to their homes, instead of taking healthy short walks to the grocery store (5). Self-directed “physically active” individuals only exercise for 45-60 minutes per session, twice or three-times per week (6). The slow but constantly forged genes of humans were not designed for such low levels of activity. In terms of evolutionary metabolic/physiological adaptations, humans were designed to move frequently, run, and fight for food, and survive in an often-unfriendly environment (7). In developed countries, the vast availability of calories in addition to sedentary lifestyles has led to a high incidence of modern life (chronic) diseases, such as obesity, diabetes, heart disease, cancers, and many other complex psychosomatic and inflammatory disorders (8).

Regular exercise is important to the general health of humans. Individual judgment and scientific data indicate that exercise is the best non-pharmacological intervention (combined with good nutrition) to assure a good quality of life at any age (9–11). Physical exercise promotes cardiovascular efficiency, activates protein and energy metabolism through progressive endocrine adjustments, optimizes immune responses, and provides cognitive benefits, especially in committed individuals who engage in regular exercise programs (12). Although the benefits of exercise are known to be dependent on the intensity and duration of the training sessions adjusted to the physiological conditions of the participants (13), understanding the cellular pathways that regulate/revert/avoid extended post-exercise inflammatory condition has become a very important target (14).

Exercise affects all organs, tissues, and cells in the human body. Several molecular events are particularly important for eliciting health-related physiological adaptations, including cellular redox rebalancing, which involves the production of reactive oxygen/nitrogen species (ROS/RNS) counteracted by antioxidant defenses (15), skeletal muscle hypertrophy (including fiber-type distinctions) (15), mitochondrial biogenesis, fission-fusion dynamics (16), angiogenesis (17), and other mechanisms (18). However, the precise cellular and molecular mechanisms that underlie the beneficial effects of exercise remain unclear. Myokines constitute a new regulatory component that may play a role in exercise-induced adaptation (16–21). In this mini review, we sought to elucidate how myokines, in particular the exerkine, irisin, act in body homeostasis and under pathological conditions.

## 2 Skeletal muscle as an excretory organ

The skeletal muscle is the most abundant tissue in the human body and is responsible to produce strength, balance, and movement (21, 22). The skeletal muscle is also known as an endocrine, paracrine, and autocrine organ that produces the myokines required for integrative responses in the human body (19, 23, 24).

The skeletal muscle releases myokines, which are signaling molecules that promote an integrative crosstalk between the muscle and other organs, resulting in physiological benefits from exercise (25–28). Physical inactivity causes chronic inflammation, mainly owing to the inappropriate release of cytokines from redox-imbalanced (or injured) tissues or overstimulation of inflammatory cells, such as neutrophils and macrophages (29). Interestingly, several exercise protocols have been proven to reduce the mortality risk of individuals with a previous sedentary lifestyle, an effect that could be directly attributed to the signaling adaptation of key myokines. For instance, interleukin (IL)-6, the first and most studied myokine, is produced *via* muscle contraction during exercise training sessions and is released in circulation to induce lipolysis in adipose tissue and glycogenolysis in the liver. IL-6 increases the expression of IL-1ra, which can be observed within 1 to 2 h after 2.5 hours of exercise at 75% VO_2max_. IL-10 levels were found to peak at 45 and 72 h after resistance training and cycle ergometer at 60% VO_2max_, and at 4 h after a marathon (30, 31). Therefore, the expression intensity and circulating levels of the myokine, IL-6, and other factors, such as tumor necrosis factor-alpha (TNF-α) and C-reactive protein (CRP) (32, 33) are associated with muscle-derived signals for energy supply and post-exercise inflammatory responses (34, 35).

A novel jargon used in clinical and sports medicine, “*exerkines*,” may better define this myokine action in response to acute or chronic exercise (20) Among several previously reported cytokines (such as IL-6), key signaling agents have been highlighted from contractile muscles, such as angiopoietin-like 4 (ANGPTL4), apelin, brain-derived neurotrophic factor (BDNF), CCL2 (or MCP-1), CX3CL1 of fractalkine (FKN), fibroblast growth factor 21 (FGF21), IL-7, IL-8, IL-15, myostatin, secreted protein acidic and cysteine-rich (SPARC), leukemia inhibitory factor (LIF), meteorin-like protein (Metrnl), and irisin (36). These cytokines have multiple effects on the body, ranging from cardiovascular functions, with proven interactions with vascular endothelial growth factor (VEGF) and nitric oxide (NO•), to their pivotal participation in the triggering of post-exercise inflammation (37–40).

The skeletal muscle releases non-coding RNAs, especially microRNAs (miRNAs), which have been identified as new regulatory components that may play a role in exercise-induced adaptations. However, the function of these RNAs in circulation remains unclear (41). miRNAs are a group of endogenous small non-coding RNAs that are 18–25 nucleotides in length. miRNAs regulate gene expression at the post-transcriptional level through messenger RNA degradation or translational inhibition (42). Once released by skeletal muscle cells, circulating miRNAs are stable, easily detectable, and may regulate gene expression in target cells and tissues as a novel mode of intercellular communication comparable to that exhibited by myokines. Accordingly, specific circulating miRNAs are altered in response to different acute and chronic exercise protocols in healthy and diseased populations (43). miRNA-133 is a muscle-enriched miRNA that regulates myogenesis *in vitro* by increasing myoblast proliferation. The expression level of miRNA-133 in skeletal muscles is sensitive to muscle contraction in response to several types of endurance exercise. As a result, miRNA-133 is a suitable candidate for potential post-exercise regulation at the plasma level (41). Therefore, exercise-induced changes in circulating miRNAs are dependent on muscle mass, angiogenesis, inflammation, ischemia, and hypoxia (44).

Of all the active exerkines, irisin has been demonstrated as one of the main protagonists in the modulation of blood pressure control *via* the NO•-dependent pathways. Moreover, irisin is expected to be a potential therapeutic agent for the treatment of non-communicable diseases and their related conditions and is regulated by the expression of some miRNAs (45). As a result, irisin is the focus of our review.

## 3 Irisin and physical exercise

Irisin is a relatively small peptide with 112 amino acids and a specific domain for the transmembrane protein, fibronectin (FNDC5). The start codon of the human FNDC5 gene is reported to be atypical ATA rather than ATG in rodents. Further, the translation efficiency of the human FNDC5 gene constructed with the ATA start codon is impaired. Other hominid species, such as Denisovan and Neanderthal, also display a loss in FNDC5 gene expression (46). Since the first discovery of irisin, scientists have tried to understand and determine the cleavage protein for FNDC5 (46). In fact, by using an improved mass spectrometry technique with synthetic peptides rich in heavy stable isotopes as internal standards, Jedrychowski et al. found that irisin is mainly expressed in the non-canonical start codon of FNDC5 (47). Based on recent studies, irisin binds to proteins of the αV class of integrins. Further biophysical studies revealed interacting surfaces between irisin and αVβ5 integrin (48). Chemical inhibition of αV integrins blocks the signaling pathway activated by irisin in both osteocytes and fat cells (49). These studies suggest the existence of the membrane receptors of irisin, as predicted by some scholars, with αVβ5 integrin as the receptor of irisin in osteocytes, adipocytes, and enterocytes (48, 49). However, the presence of irisin receptors in other cells requires further studies. In addition, the results of the irisin assays must be interpreted with caution as after ten years, the baseline values for western blotting and ELISA are associated with a large variance and problems with reproducibility may arise among lots (46, 50).”

In adipose tissue, FNDC5 exhibits its activity *via* uncoupling protein type-1 (UCP-1), which promotes the so-called “browning” process of adipose tissue. This activity is associated with an increase in the mitochondrial-rich adipocyte population within the fat tissue, ultimately leading to increased heat production and energy expenditure by these cells (51, 52). Interestingly, some miRNAs, such as miRNA-19b and miRNA-140, are suggested to downregulate irisin expression, thereby promoting weight loss by reducing energy expenditure (53, 54).

Irisin is produced in various tissues. In animals, irisin production was identified in the muscle, liver, pancreas, lung, adrenal glands, central nervous system (CNS), and kidney (53), whereas in humans, its production was identified in other tissues, such as adipose, bone, cardiomyocytes, and sebaceous glands. However, contracting skeletal muscles are the main sources of irisin production in the human body (54). In addition to its ability to induce mitochondrial biogenesis, irisin can regulate oxidative metabolism in different cell types *via* autocrine, paracrine, and endocrine mechanisms (55, 56). Notably, ROS/RNS production, and consequently the redox status in active skeletal muscles, is influenced by irisin activity (57, 58).

Skeletal muscle contraction induces the transcription and activation of the transcription coactivator, peroxisome proliferator-activated receptor gamma (PPAR-γ) type 1 alpha (PPARGC1A, also known as PGC-1α), a master regulator of genes involved in metabolism. The overexpression of PGC-1α increases irisin production *via* cleavage of the FNDC5 factor, stimulating mitochondrial biogenesis, oxidative phosphorylation, and oxygen consumption rate (55, 56). In response to exercise, irisin regulation depends on the specific training protocol (intensity, duration, and type of exercise), age, sex, training status, and muscle mass. Short bouts of intensive exercise acutely increase serum irisin levels in children and adults; however, irisin levels do not differ following prolonged (6 weeks) or chronic (1 year) exercise (57).

The activity of the FNDC5/irisin protein activates MAP-kinase cascades, and upon activation, the differentiation of neural cells and osteocytes is achieved, concomitant with the enhancement of glucose uptake by muscles and subsequent “browning” of white adipose tissues (58). Among the several MAPK pathways, the most conventional routes are c-Jun N-terminal kinases 1-3 (JNK1-3), extracellular signal-regulated kinase 1 and 2 (ERK1/2), p38 isoforms (α, β, γ, and δ), and ERK5 families. Less understood pathways, such as ERK3/4 and ERK7/8, and stress-activated protein kinases (SAPK1A, 1 B, 1C) (59, 60). Zhang et al. (2014) showed that chemical inhibition of the p38 or ERK pathways causes a significant reduction in the action of irisin on the adipocyte UCP-1 protein (61).

The exercise-induced irisin effect can differ in middle-aged, older, and young adults. Miyamoto-Mikami et al. showed that 8-weeks of endurance training promoted higher serum irisin expression in healthy middle-aged and older people (65 ± 8 years old) but not in young people (21 ± 1 years old). In contrast, younger and older adults displayed a similar irisin response to an acute bout of circuit training. Irisin levels were also found to have a significant correlation with a less visceral adipose tissue but not with whole-body fat mass (62) In a recent systematic review and meta-analysis, exercise training was found to significantly increase circulating irisin, and decrease insulin, glucose, and insulin resistance. Notably, the employed exercise training protocol may be associated with different irisin expression, suggesting that irisin level significantly increased when resistance training and resistance training combined with aerobic training were applied, while insulin level decreased when aerobic training and combined training were employed, especially in patients with type 2 diabetes and prediabetes (63). In another study, different muscles and training types were compared among mice. Based on the results, the slow-twitch muscle produced more irisin than the fast-twitch muscles; however, the training type (aerobic or anaerobic) did not affect irisin production (64)

Beyond its pivotal role in energy metabolism and mitochondrial biogenesis, FNDC5/irisin exerts inhibitory effects on inflammation by inducing hyperphosphorylation of MAPKs and reducing the release of pro-inflammatory cytokines (65). In addition, the association between irisin and nervous system function has been revealed during neural differentiation, when the loss of ERK1/2 function causes a significant decrease in the expression of both FNDC5/irisin and BDNF (66).

**Figure 1:**
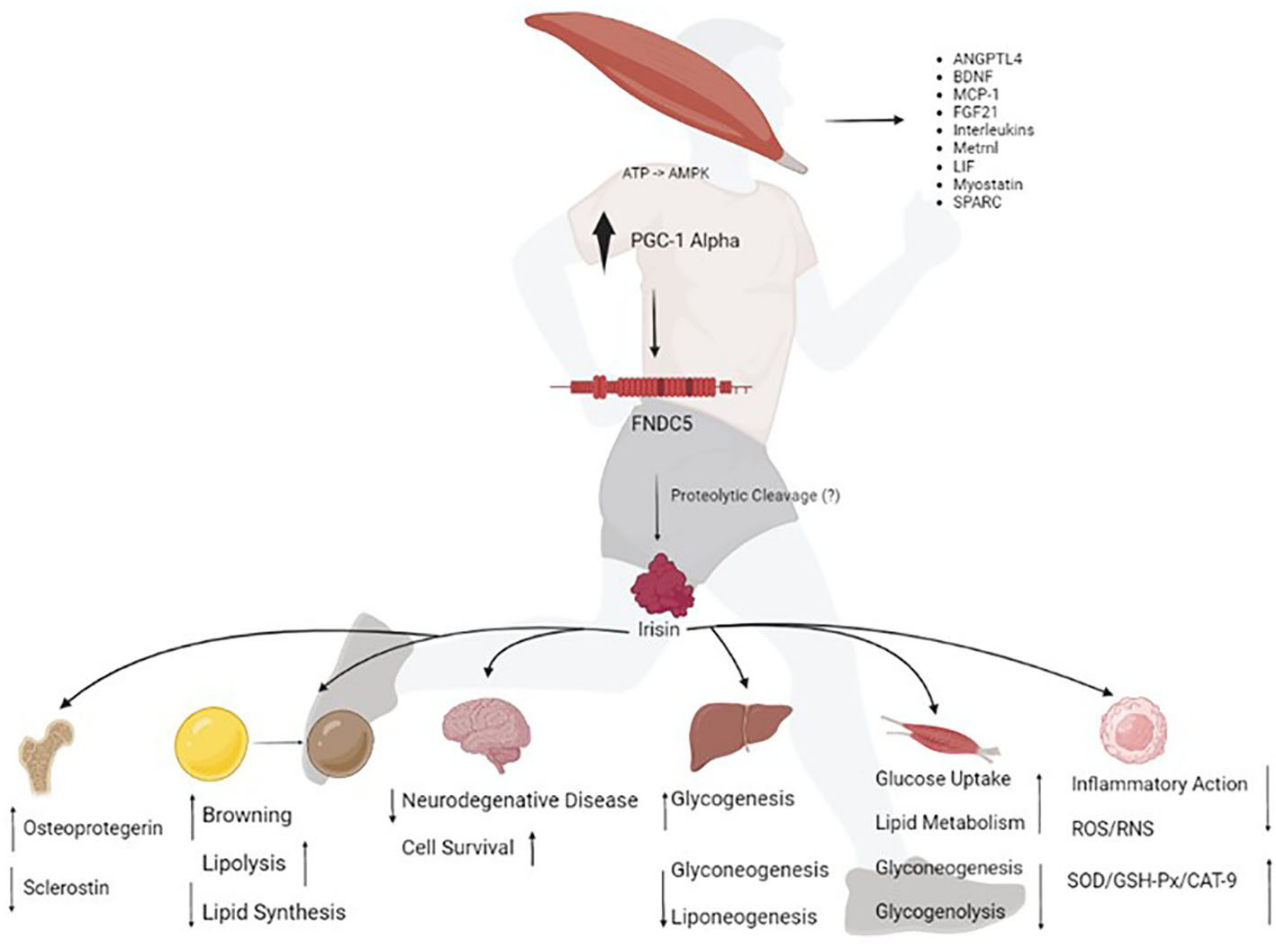
The exercise stimulates the peroxisome proliferator-activated receptor gamma coactivator 1 (PCG1)-alpha transcription, which in turn drives the expression of fibronectin type III domain-containing protein 5 (FNDC5), a membrane protein that is cleaved and secreted as irisin. Irisin acts on various human organs and tissues; which together orchestrate whole-body metabolism by regulating bone remodeling, "browning" of mature white adipocytes in response to exercise, glucose metabolism and insulin sensitivity in skeletal muscle, neuroplasticity, insulin sensitivity, and improving hepatic glucose and lipid metabolism. Irisin improves redox balance and inflammation. ATP, adenosine triphosphate; AMPK, AMP-activated protein kinase; ANGPTL4, angiopoietin-like 4; BDNF, brain derived neurotrophic factor; MCP-1, monocyte chemoattractant protein-1; FGF21, fibroblast growth factor 21; LIF, leukemia inhibitory factor; Metrnl, meteorin-like protein; SPARC, secreted protein acidic and cysteine-rich; ROS, reactive oxygen species; RNS, reactive nitrogen species; SOD, dismutase superoxide; GSH-PX, glutathione peroxidase and CAT-9, catalase.

Overall, FNDC5/irisin can act as an important regulator of cellular communication, especially between muscle and other tissues. As suggested by Maalouf and Khoury (2019), FNDC5/irisin serves as a remarkable potential pharmacological target for inflammation and energy metabolism control (67). **Figure 1** summarizes the effects of irisin on several organs and tissues.

## 4 Irisin, inflammation, and illness

Obesity and type-2 diabetes mellitus are accompanied by the upregulation of inflammatory markers that substantially contribute to an overproduction of ROS/RNS, favoring insulin resistance in peripheral tissues and impaired insulin secretion from the pancreas (68, 69). As previously mentioned, irisin has a prominent capacity to modulate peripheral energy metabolism. Further, higher systemic irisin levels have been demonstrated to ameliorate glucose tolerance and mitigate insulin resistance (70). These effects of irisin can be observed through the upregulation of thioredoxin 2 (Trx2) and the blocked expression of thioredoxin-interacting protein (Txnip), consequently reducing the number of β-cells. Accordingly, irisin administration can help us understand its possible protective effect (71). Irisin was demonstrated to stimulate glucose uptake in skeletal muscle cells by increasing the phosphorylation of AMPK, thereby activating p38, which leads to the translocation of GLUT4 from the perinuclear region to the plasma membrane (72). This antidiabetic effect might be related to the ability of exercise-derived irisin to reduce or modulate inflammation. The anti-inflammatory action of irisin is reported to be particularly prominent in circulating immune cells, such as macrophages and neutrophils. Similarly, Mazur-Bialy et al. (73) demonstrated that irisin not only improves macrophage phagocytosis but also reduces ROS/RNS production in activated immune cells, which has a remarkable impact on inflammatory responses. The same researchers reinforced that the anti-inflammatory properties of irisin could be attributed to its ability to downregulate the downstream pathways of TLR4/MyD88 in RAW 264.7 macrophages (65, 73). The researchers also demonstrated the ability of irisin to inhibit the expression and release of inflammatory mediators in a coculture of adipocytes and macrophages (73). These researchers also showed that the Nf-r2/Heme oxygenase-1 (HO-1) pathways can be stimulated, consequently leading to less effects of ROS in the cell. Exercise promotes higher expression of NO, a factor that can stimulate HO-1; therefore, the antioxidant effects could be related to the Nf-r2/HO-1 pathways in the system (74). Treatment with irisin was found to stimulate antioxidants (SOD, GSH-Px, and CAT-9), ultimately reducing the levels of H_2_O_2_ in macrophages *in vitro (* 75).

Interestingly, some studies have already revealed that irisin can reduce the progression of inflammation associated with inflammatory bowel disease (IBD) by reducing pro-inflammatory cytokine release (76, 77). IBD is related to reduced bone mineral density (DMO) induced by inflammation, and treatment with irisin induces bone formation and reduces the TNF-α^+^. Thus, irisin could reduce bowel inflammation and sclerostin (SOST) production, and reinvigorate DMO through osteoprotegerin (OPG) stimulation (46, 77). The volume intensity suggested a moderate intensity (60% VO_2max_) for 1 has no gastrointestinal changes were observed and intestinal inflammation was reduced (78) More studies are needed to delimit an exercise protocol for the treatment of these patients. Beyond these remarkable findings, higher levels of exercise-derived irisin can induce the expression of several anti-inflammatory proteins in the brain, particularly BDNF, in the hippocampus, a well-known brain segment associated with memory. Overall, irisin has putative applications in reverting the cognitive decline associated with Alzheimer’s disease (already demonstrated using experimental models) through exercise (79).

In a previous study, hepatic-released irisin was demonstrated to function as a paracrine/autocrine factor that inhibits lipogenesis and gluconeogenesis *via* the adenosine 5’-monophosphate (AMP)-activated protein kinase pathway by reducing the expression of phosphoenolpyruvate carboxyinase (Pepck) and glucose-6-phosphate (G6P) genes (80, 81). Accordingly, irisin could be a new treatment for the diabetic population.

In summary, both *in vivo* and *in vitro* studies revealed that irisin has remarkable anti-inflammatory properties as it modulates cytokine production, induces MAPK cascade factors (NF-kB), and reduces oxidative stress in different contexts. However, further mechanistic studies directly addressing the effects of irisin on ROS/RNS production and antioxidant defenses are needed.

## 5 Irisin and aging

Irisin is a myokine linked to many age-related diseases and neurological disorders (82–89). Studies in gerontology demonstrated that the pro-inflammatory response of adipose tissue contrasts with the anti-inflammatory action of exercise-induced myokines, such as irisin, released by contractile muscle tissue (23, 90). Unfortunately, only few studies have combined these variables into the same model. In 2020, Rashid et. al. analyzed the response of irisin to long-term moderate physical exercise (91). Their findings highlighted the influence of long-term exercise on metabolic mediators. After long-term physical exercise, irisin was demonstrated to improve glucose homeostasis, which was correlated with better glucose regulation, less insulin resistance, and consequently obesity. The protocol was tested in two groups: normal weight group (BMI < 25 kg/m^2^) and obese group (BMI ≥ 30 kg/m^2^). Notably, resistance training was adopted in this study. As aging comorbidities were consistently supported by the physiological pattern of an unhealthy aging lifestyle (87, 88), these studies could be perceived as early explorations into the positive effects of exercise-regulated irisin levels in the elderly population. To the best of our knowledge, this study is the only report of this information.

Numerous data have suggested the putative effect of increased irisin levels on reverting or retarding obesity and aging progression, based on renowned biomarkers, such as glycemia and total cholesterol levels (92). Previous studies have shown that irisin can act as a regulatory factor to control diabetes and obesity during aging (93).

Obesity and diabetes can increase the risk of cognitive ageing (94) and dementia (95). These cognitive conditions represent a significant challenge for the scientific and clinical communities (14). Exercise can have beneficial effects on brain health (9–11). Based on strong evidence, irisin plays a crucial role in the cognitive benefits of exercise. Prior studies also shed light on irisin as a potential therapeutic agent for some cognitive disorders (88, 96, 97). Cognitive function was previously thought to be improved by the increased expression of BDNF, which is stimulated by exercise-induced irisin and lactate (98, 99). Diabetes may also induce neuroinflammation, and consequently, cognitive deterioration and reduced memory. Treatment with irisin was found to block the p38, STAT3 and Nf-κB proteins, reducing diabetes-induced neuroinflammation in the brain of mice (100) Another study revealed that 100 nm/L irisin promoted cell proliferation *via* the STAT3 pathway (101). Synaptogenesis, neurogenesis, and long-term potentiation can be molecular pathways that explain the BDNF-mediated improvement in neuroplasticity *via* irisin (102). Moreover, a higher expression of BDNF affects the levels of dopamine, serotonin, and melatonin in several brain regions, preventing many of the symptoms observed in individuals with depression (65) and directly benefitting learning and memory *via* the dopamine pathways (103). When BDNF is stimulated by lactate (via SIRT1 pathway activation), memory and learning functions are substantially improved compared with instances of lower lactate concentrations (98). Notably, such findings were transversal and enthusiastically received from the psychological scientific community. However, lactate levels may reflect exercise intensity, which may be mainly responsible for the reported associations with improved memory function. Other benefits of exercise on brain function and the prevention of dementia include improved cerebral perfusion, improved metabolism (104), and reduced inflammation.

Immunological analysis must be performed to understand how the MAPK, AMPK, and TLR4/MyD88 intercellular pathways and exercise-induced irisin can benefit systemic inflammatory conditions (105). Recently, Papadopoulos, et al. reported that irisin released by muscles during aerobic exercise is an active agent in the AMPK/Akt-eNOS/NO• pathway (106).

**Figure 2:**
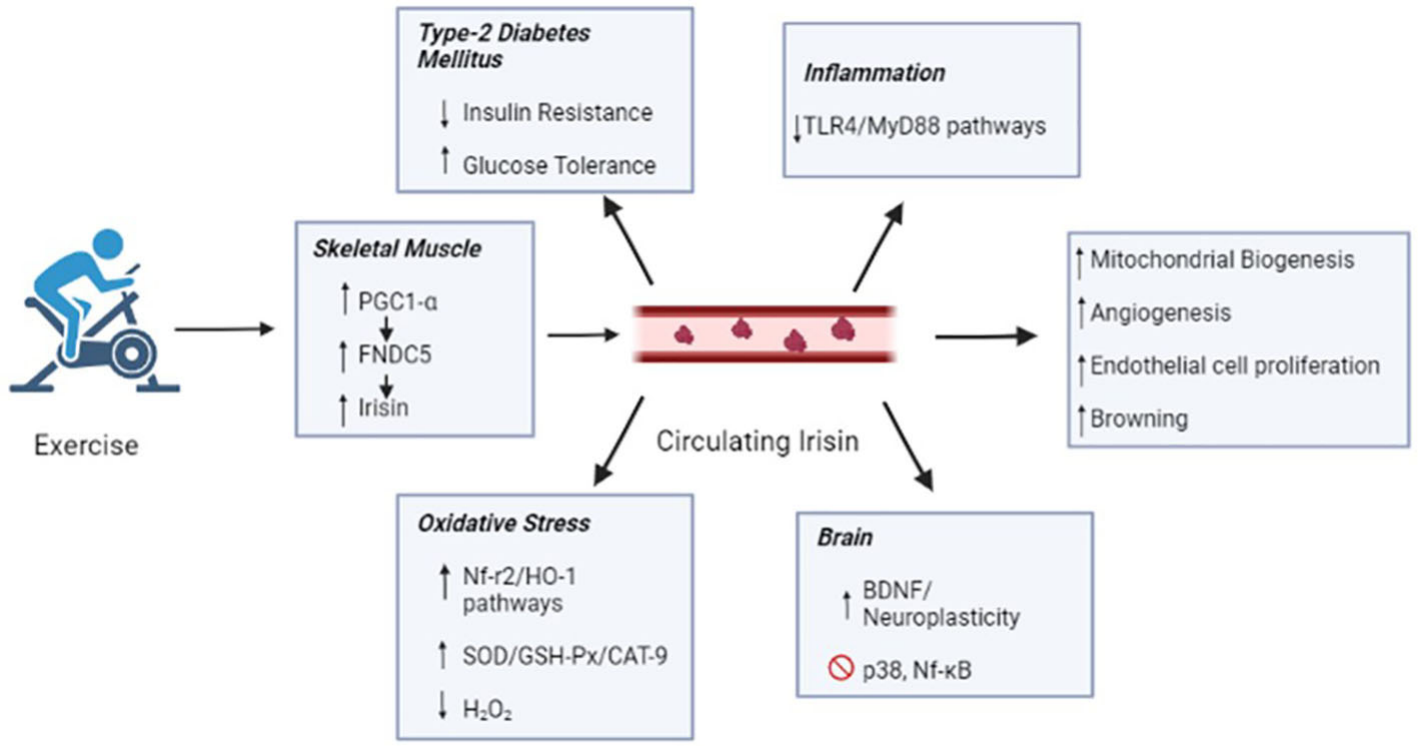
Irisin secreted by skeletal muscle during the exercise reaches blood circulation and in general ameliorates the mitochondrial biogenesis, angiogenesis, endothelial cell proliferation and browning. Such benefits might explain the protective effects of exercise against type-2 Diabetes Mellitus, inflammation, oxidative stress and neurodegenerative disease. PGC1-α, peroxisome proliferator-activated receptor gamma coactivator 1 alpha; FNDC5', fibronectin type III domain-containing protein 5; TLR4, toll-like receptor 4; MyD88, myeloid differentiation primary response 88; HO-1, heme oxygenase-1; SOD, dismutase superoxide; GSH-Px, peroxidase glutathione; CAT-9, catalase; H202, oxygen peroxide; BDNF, brain-derived neurotrophic factor and NF-kB, factor nuclear kappa B.

Overall, the current evidence reinforces the hypothesis that irisin at optimal levels could be the main agent responsible for the long-term health benefits in regularly exercising individuals. Acute exercise can increase the concentration of circulating irisin, while chronic exercise can improve irisin metabolic dynamics and selectively increase circulating irisin concentrations. The effects of irisin may mediate some beneficial effects of exercise, such as the enhanced oxidation of fatty acids and heat production, leading to increased energy expenditure, glucose homeostasis, weight reduction, mitochondrial biogenesis, angiogenesis, improved cognition function, muscle fiber shifting, and prevention of muscular atrophy in aging and metabolic diseases (**Figure 2**). These effects can be regulated by distinct molecular pathways that permeate redox signaling and miRNA-mediated ncRNAs. However, the exact mechanisms remain unclear as the available data are barely consistent. Therefore, it is important to highlight how irisin can be used in the future as a diagnostic tool and possible treatment for the population. Further studies are necessary based on the metabolic, physiological, and cognitive limitations imposed by aging and pathological processes.

## 6 Conclusions

Herein, we addressed the mediators elicited during exercise for the maintenance of good health. We focused on the myokines produced by contracting muscles, which are also known as exerkines. Some of these mediators, including miRNAs, are released into the circulation, thereby improving cell-to-cell communication. Herein, the role of irisin in metabolism and inflammation was revealed, including its subsequent effects on obesity and diabetes mellitus, cognitive function, and compromised immune function. Further studies are necessary to demonstrate the possibility of biomarker-led diagnostics, conduct further mechanistic assessments, and identify novel treatment regimes.

## Author contributions

Conceptualization, CT, BP, MB, and TF. writing, CT, BP, MB, AB, PB, CM, GF, PV. writing—review and editing, CT, BP, MB, AB, PB, CM, GF, PV, EO, EH and TF. visualization, CT, BP, MB, AB, PB, CM, GF, PV, EO, EH and TF. supervision, TF. All authors have read and agreed to the published version of the manuscript. All authors contributed to the article and approved the submitted version.
